# Predictors of mortality and morbidity in critically ill COVID‐19 patients: An experience from a low mortality country

**DOI:** 10.1002/hsr2.542

**Published:** 2022-05-17

**Authors:** Mohamad Y. Khatib, Dore C. Ananthegowda, Moustafa S. Elshafei, Hani El‐Zeer, Wael I. Abdaljawad, Muhsen A. Shaheen, Abdulsalam S. Ibrahim, Ahmad A. Abujaber, Ahmed A. Soliman, Ahmed S. Mohamed, Mohammad Al‐Wraidat, Amna Ahmed, Abdulqadir J. Nashwan, Mohamed O. Saad, Adeel A. Butt, Muna A. Al‐Maslamani, Ahmed Al‐Mohammed

**Affiliations:** ^1^ Department of Medicine, Division of Critical Care Hazm Mebaireek General Hospital, Hamad Medical Corporation Doha Qatar; ^2^ Department of Medicine, Division of Critical Care Hamad General Hospital, Hamad Medical Corporation Doha Qatar; ^3^ Division of Critical Care Nursing Hazm Mebaireek General Hospital, Hamad Medical Corporation Doha Qatar; ^4^ Pharmacy Department Al Wakra Hospital, Hamad Medical Corporation Doha Qatar; ^5^ Department of Medicine Hamad Medical Corporation Doha Qatar; ^6^ School of Medicine Qatar and Weill Cornell Medicine Ar‐Rayyan Qatar; ^7^ School of Medicine Qatar and Weill Cornell Medicine New York New York USA; ^8^ Department of Infectious Disease Communicable Diseases Centre, Hamad Medical Corporation Doha Qatar

**Keywords:** ARDS, COVID‐19, intensive care, mortality, SARS‐CoV‐2, Qatar

## Abstract

**Background and Aims:**

Clinical characteristics and factors associated with mortality in patients admitted to the intensive care unit (ICU) in countries with low case fatality rates (CFR) are unknown. We sought to determine these in a large cohort of critically ill COVID‐19 patients in Qatar and explore the early mortality predictors.

**Methods:**

We retrospectively studied the clinical characteristics and outcomes in patients admitted to the ICU at the national referral hospital for COVID‐19 patients in Qatar. Logistic regression analysis was used to determine factors associated with mortality.

**Results:**

Between March 7 and July 16, 2020, a total of 1079 patients with COVID‐19 were admitted to the ICU. The median (IQR) age of patients was 50 (41–59) years. Diabetes (47.3%) and hypertension (42.6%) were the most common comorbidities. In‐hospital mortality was 12.6% overall and 25.9% among those requiring mechanical ventilation. Factors independently associated with mortality included older age ([OR]; 2.3 [95% CI; 1.92–2.75] for each 10‐year increase in age, *p* < 0.001), chronic kidney disease (OR; 1.9 [95% CI; 1.02–3.54], *p* = 0.04), active malignancy (OR; 6.15 [95% CI; 1.79–21.12], *p* = 0.004), lower platelet count at ICU admission (OR; 1.41 [95% CI; 1.13–1.75] for each 100 × 103/µl decrease, *p* = 0.002), higher neutrophil‐to‐lymphocyte ratio at admission (OR; 1.01 [95% CI; 1–1.02] for each 1‐ point increase, *p* = 0.016), higher serum ferritin level at admission (OR; 1.05 [(95% CI; 1.02–1.08] for each 500 µg/L increase, *p* = 0.002), and higher serum bilirubin level at admission (OR; 1.19 [95% CI; 1.04–1.36] for each 10 μmol/L increase, *p* = 0.01).

**Conclusions:**

The mortality rate among critically ill COVID‐19 patients is low in Qatar compared to other countries. Older age, chronic kidney disease, active malignancy, higher neutrophil‐to‐lymphocyte ratios, lower platelet counts, higher serum ferritin levels, and higher serum bilirubin levels are independent predictors of in‐hospital mortality.

## 1 INTRODUCTION

Coronavirus disease‐19 (COVID‐19) primarily affects the respiratory system, though numerous other organs may be involved.^1^ Case fatality rate (CFR) varies significantly among different countries ranging from as low as 0.05% in Singapore to around 8% in Mexico.^2^ Higher mortality in persons with confirmed COVID‐19 is associated with older age and the presence of comorbidities, such as diabetes mellitus type 2, hypertension, and impaired kidney function.^1^, ^2^ However, demographic differences and the burden of comorbidities likely do not fully account for the vastly different case fatality rates in various countries, and other hosts, pathogen, or environmental factors may play a role. Access to care, particularly early hospital and intensive care unit (ICU) admission are plausible hypotheses that may affect mortality in these patients.

The first case of SARS‐CoV‐2 infection in Qatar was diagnosed on February 28, 2020, in a returning traveler. The first locally acquired case was diagnosed on March 7, 2020. Qatar has one of the highest infection rates per capita in the world but has also witnessed one of the lowest case fatality rates.^2^ With a population of approximately 2.8 million persons, Qatar has recorded only 246 deaths among 145,672 patients (0.17%) by October 2021.^3^ Patients who require intensive care have significantly higher mortality, ranging from 26% to 97% in various countries.^4^, ^5^, ^6^, ^7^, ^8^, ^9^, ^10^, ^11^ While demographic characteristics and the presence of comorbidities are associated with higher mortality in patients admitted to the ICUs, the wide variation in mortality rate is not fully explained by these differences alone. These varied case fatality rates, for the same disease COVID‐19, are probably not only because of the variance by virus or host but because of various environmental factors like the sudden surge of cases and nonavailability of resources and early access to critical care for all deserving cases. Our current knowledge is derived largely from countries with a high case fatality rate and predictors of mortality may be different in countries with a low case fatality rate.

In this study, we aim to describe the clinical characteristics and the complete outcomes of a large cohort of critically ill COVID‐19 patients from a national referral hospital of Qatar and to explore the early predictors of in‐hospital mortality among this cohort.

## 2 MATERIALS AND METHODS

### 2.1 Settings

This study was conducted at Hazm Mebaireek General Hospital (HMGH), the main referral center for COVID‐19 treatment in Qatar. We retrospectively analyzed the data of patients with reverse transcriptase‐polymerase chain reaction (RT‐PCR) confirmed SARS‐CoV‐2 infections who needed admission to the ICU between March 7 and July 16, 2020. The outcome data were collected up to August 18, 2020. The study conformed to the Declaration of Helsinki principles and was approved by the ethics committee of the Hamad Medical Corporation Medical Research Centre. HMGH was a newly built hospital that just opened for admissions before the COVID‐19 outbreak and then was converted into a completely dedicated critical care center during the pandemic. This process involved ramping up the total number of ICU beds from 8 to 226. This was implemented with meticulous planning to create structured ICU units well‐staffed with appropriately trained providers. Our treatment protocol included hydroxychloroquine, oseltamivir, ritonavir plus lopinavir or remdesivir at later stages, and antibiotics if indicated plus methylprednisolone or dexamethasone and anticoagulants based on local guidelines. Few selected patients received convalescent plasma transfusion. Acute respiratory distress syndrome (ARDS) was managed with high‐flow nasal cannula, noninvasive ventilation, or invasive ventilation with prone ventilation if required and extracorporeal membrane oxygenation (ECMO) in refractory cases. Tocilizumab was also administered in selected few cases when interleukin‐6 levels were high.

### 2.2 Inclusion/exclusion criteria

We included adult patients (18 years of age or older) admitted to HMGH ICU with a confirmed diagnosis of COVID‐19 and complete outcome data, either discharge from hospital or mortality. Patients who stayed for less than 24 h in the ICU were excluded. ICU stay of at least 24 h is used to show any meaningful interventions from critical care which altered patients outcomes; if they die less than 24 h, it is probably more because of the ineffective support from prehospital care or already very sick at admission, or if discharged within 24‐h, likely that they did not require ICU care at all from the beginning, hence 24‐h cutoff limit.

### 2.3 Data collection

Patient data, including sociodemographic information, clinical data, and laboratory data, were obtained from the electronic medical records. The primary outcome was in‐hospital mortality. We collected data on the incidence of complications including venous thromboembolism, rhabdomyolysis, secondary infection and bleeding, and the requirement of organ support including invasive mechanical ventilation (IMV), vasopressor therapy, renal replacement therapy (RRT), and ECMO. Tracheostomy procedures were also captured.

### 2.4 Statistical analysis

Qualitative data are presented as counts with percentages and compared using the *χ* ^2^ test or Fisher's exact test, while quantitative data are presented as medians with interquartile range compared using the Mann–Whitney test. Potential predictor variables were selected based on the previous literature. Eligible variables were then included in the multivariable logistic regression model. Backward elimination was used for selecting significant predictors. A cutoff *p* value of more than 0.1 was used to remove variables from the initial multiple regression model to reach the final model. Two‐sided *p* values of less than 0.05 were considered statistically significant. Multicollinearity was checked using a correlation matrix and variance inflation factors. Multiple imputations procedure was used for missing values (33 values of body mass index and three values of C‐reactive protein). To assess the robustness of the mortality predictors, we repeated the analysis among male patients only as they represent the majority of the study cohort. All analyses were performed using Stata MP/16.0 Software (Stata).

## 3 RESULTS

During the study period, 1082 cases were admitted to ICU with COVID‐19 and three were excluded as the length of stay in ICU was less than 24 h, hence we retrospectively analyzed the data of the remaining 1079 patients. Overall, in‐hospital mortality was 12.6% (136/1079). The median (IQR) age was 50 (41–59) years. Most (94.4%) were men. Due to the demographics peculiar to our country, we have a large male population reflected by the number of proportionate admissions. Qatar is a middle east country with a large population of foreign workforce, predominantly males. The female population consists of the local female patients and the workers whose families live here. Most workers are single, and their families are back in their native country. Diabetes and hypertension were the most common comorbidities (47.3% and 42.6%, respectively). Overall, patients had a median PaO_2_/FiO_2_ ratio (Partial pressure of arterial oxygen/Fraction of inspired oxygen) of 129 (IQR; 98–151), median d‐dimer of 1 mg/L fibrinogen‐equivalent units (FEU) (IQR; 0.57–2.8 mg/L FEU), median serum ferritin of 1024 µg/L (IQR; 558–1616 µg/L) and median neutrophil‐to‐lymphocyte ratio (NLR) of 8 (IQR; 4.5–13.8) upon admission to the ICU (Table 1).

**Table 1:** Baseline characteristics of the study cohort (*N* = 1079)

	Survivors (*n* = 943)	Non‐survivors (*n* = 136)	*p* value	Total (*n* = 1079)
Age, years	48 (40–57)	61 (53–70)	<0.001	50 (41–59)
Male	890 (94.4%)	127 (93.4%)	0.64	1017 (94.3%)
BMI, kg/m^2^	27.11 (24.49–30.48)	27.4 (24.70–30.90)	0.36	27.18 (24.50–30.50)
Diabetes	436 (46.2%)	74 (54.4%)	0.074	510 (47.3%)
Hypertension	376 (39.9%)	84 (61.8%)	<0.001	460 (42.6%)
Coronary artery disease	122 (12.9%)	25 (18.4%)	0.084	147 (13.6%)
Chronic kidney disease	62 (6.6%)	28 (20.6%)	<0.001	90 (8.3%)
Chronic lung disease	46 (4.9%)	12 (8.8%)	0.056	58 (5.4%)
Dyslipidaemia	88 (9.3%)	18 (13.2%)	0.15	106 (9.8%)
Chronic liver disease	12 (1.3%)	7 (5.1%)	0.001	19 (1.8%)
Active malignancy	8 (0.8%)	8 (5.9%)	<0.001	16 (1.5%)
SOFA score	2 (2–3)	3 (2–5)	<0.001	2 (2–3)
Glasgow Coma Scale	15 (15–15)	15 (15–15)	0.002	15 (15–15)
PaO_2_/FiO_2_ ratio, mmHg	129 (99–152)	125 (96–140)	0.057	129 (98–151)
Ferritin, µg/L	1008 (547–1570)	1113 (650–2353)	0.013	1024 (558–1616)
d‐dimer, mg/L FEU	0.94 (0.53–2.59)	1.77 (0.81–5.22)	<0.001	1 (0.57–2.80)
C‐reactive protein, mg/L	123.9 (59.2–218)	157.5 (90.4–255.2)	0.004	128.1 (63–223.5)
NLR	7.7 (4.4–12.7)	12.21 (5.5–19.4)	<0.001	8 (4.5–13.8)
Platelet count (×103/µl)	259 (201–331)	226.5 (161–287)	<0.001	253 (197–326)
Bilirubin, μmol/L	10 (7–15)	11 (7–18)	0.22	10 (7–15)
Creatinine, μmol/L	79 (65–97)	93.5 (70–139)	<0.001	80 (66–101)

*Note*: Continuous data are presented as median (interquartile range) and categorical data are presented as frequency (percentage).

Abbreviations: BMI,  body mass index; PaO_2_/FiO_2_ ratio, arterial oxygen partial pressure divided by the fraction of inspired oxygen; SOFA, sequential organ failure assessment.

Compared to survivors, nonsurvivors were older (median age [IQR]; 61 [53–70] vs. 48 [40–57] years, *p* < 0.001) and more likely to have hypertension (61.8% vs. 39.9%, *p* < 0.001) and chronic kidney disease (CKD) (20.6% vs. 6.6%, *p* < 0.001). Nonsurvivors also had higher values of d‐dimer (median [IQR]; 1.77 [0.81‐5.22] vs. 0.94 [0.53‐2.59] mg/L FEU, *p* < 0.00), C‐reactive protein (CRP) (median [IQR]; 157.5 [90.45–255.25] vs. 123.9 [59.20–218] mg/L, *p* = 0.004), serum ferritin (median [IQR]; 1113 [650–2353] vs. 1008 [547–1570] µg/L, *p* = 0.013), and NLR (median[IQR]; 12.21 [5.5–19.4] vs. 7.7 [4.4–12.7], *p* < 0.001). Nonsurvivors had lower platelet counts (median [IQR]; 226.5 [161–287] vs. 259 [201–331] × 10^3^/µl, *p* = <0.001), compared to survivors (Table 1).

Corticosteroids were administered in 85.6% of the study cohort. All except three patients (99.7%) received anticoagulation therapy according to our local protocol. Convalescent plasma was given to 21.6% of the patients, while tocilizumab was given to 46.9% of the patients. Among the clinical interventions provided to the patients, 521 (48.3%) of patients required IMV. Among patients who required IMV, the mortality was 25.9%. The median duration of mechanical ventilation among survivors was 6 days (IQR; 3–10 days). ECMO was initiated in 25 (2.3%) patients, while RRT was required in 129 (12%) patients.

Compared to survivors, non‐survivors were more likely to receive vasopressors (97.1% vs. 35.9%, *p* < 0.001), IMV (99.3% vs. 40.9%, *p* < 0.001), RRT (65.4% vs. 4.2%, *p* < 0.001), or ECMO (9.6% vs. 1.3%, *p* < 0.001). Additionally, the use of therapies for COVID‐19, including corticosteroids, was higher among nonsurvivors (Table 2).

**Table 2:** Clinical interventions provided to the patients during their critical illness

Intervention	Survivors	Nonsurvivors	*p* value	Total
	(*n* = 943)	(*n* = 136)		(*n* = 1079)
Corticosteroids	793 (84.1%)	131 (96.3%)	<0.001	924 (85.6%)
Anticoagulation	942 (99.9%)	134 (98.5%)	0.043	1076 (99.7%)
Iloprost	61 (6.5%)	18 (13.3%)	0.004	79 (7.3%)
Tocilizumab	433 (45.9%)	73 (53.7%)	0.09	506 (46.9%)
Convalescent plasma	188 (19.9%)	45 (33.1%)	<0.001	233 (21.6%)
Vasopressors	339 (35.9%)	132 (97.1%)	<0.001	471 (43.7%)
Renal replacement therapy	40 (4.2%)	89 (65.4%)	<0.001	129 (12.0%)
Invasive mechanical ventilation	386 (40.9%)	135 (99.3%)	<0.001	521 (48.3%)
Neuromuscular blocking agents	358 (38.0%)	126 (92.6%)	<0.001	484 (44.9%)
ECMO	12 (1.3%)	13 (9.6%)	<0.001	25 (2.3%)
Tracheostomy	32 (3.4%)	27 (19.9%)	<0.001	59 (5.5%)

Abbreviation: ECMO, extracorporeal membrane oxygenation.

VTE was diagnosed in 44 (4.1%) patients, of whom 12 (8.8%) patients died. Rhabdomyolysis was diagnosed in 189 (17.5%) patients and microbiologically confirmed secondary infections were diagnosed in 424 (39.3%) patients. Secondary infections were highest among patients who received corticosteroids and tocilizumab (48.5%), followed by patients who received corticosteroids only (38.3%) and those who received tocilizumab only (21.7%), compared to those who did not receive either (12.1%). The most common sources of infection were pneumonia (76%) followed by urinary tract infections (19%). The type of infection was bacterial in 34% of patients, fungal in 22% of them, and combined bacterial and fungal in 44% of them.

Among survivors, the median (IQR) length of ICU stay was 8 (4–13) days, while the median (IQR) hospital length of stay was 19 (14–28) days. While in deceased patients, the median (IQR) length of ICU stay was 22 (14–32.5) days, while the median (IQR) hospital length of stay was 25 (17–36) days.

In multiple logistic regression analysis, independent predictors of mortality included CKD (odds ratio [OR]; 1.9 [95% CI; 1.02–3.54], *p* = 0.044) active malignancy (OR; 6.15 [95% CI; 1.79–21.12], *p* = 0.004), older age (OR; 2.3 [95% CI; 1.92–2.75] for each 10‐year increase in age, *p* < 0.001), higher NLR at ICU admission (OR; 1.01 [95% CI; 1–1.02] for each one‐point increase in NLR, *p* = 0.016), lower platelet count at ICU admission (OR; 1.41 [95% CI; 1.13–1.75] for each 100 × 10^3^/µl decrease in platelet count, *p* = 0.002), higher serum ferritin level at ICU admission (OR; 1.05 [95% CI; 1.02–1.08] for each 500 µg/L increase in ferritin, *p* = 0.002), and higher serum bilirubin level at ICU admission (OR; 1.19 [95% CI; 1.04–1.36] for each 10 μmol/L increase in bilirubin, *p* = 0.01) (Table 3). On the other hand, the presence of coronary artery disease (CAD) was independently associated with lower risk of mortality (OR; 0.5 [95% CI; 0.27– 0.9], *p* = 0.022). Among male patients, early predictors of mortality were malignancy, older age, higher NLR at ICU admission, lower platelet count at ICU admission, higher serum ferritin level at ICU admission, and higher serum bilirubin level at ICU admission (Supporting Information Appendix 1).

**Table 3:** Early predictors of mortality among the study cohort

Predictors	Initial full model		Final model		
	OR (95% CI)	*p* value	OR (95% CI)	*p* value	VIF
Male sex	2.56 (0.94–6.97)	0.066	2.37 (0.93–6.05)	0.072	1.08
Age (per 10 years increase)	2.26 (1.87– 2.74)	<0.001	2.3 (1.92–2.75)	**<0.001**	1.19
BMI (per 1 kg/m^2^)	1.03 (0.99–1.07)	0.175	‐	‐	
Diabetes	1.05 (0.67–1.65)	0.829	‐	‐	
Hypertension	1.26 (0.78–2.05)	0.349	‐	‐	
Coronary artery disease	0.47 (0.25–0.88)	0.019	0.5 (0.27–0.90)	**0.022**	1.14
Chronic kidney disease	1.59 (0.75–3.38)	0.229	1.9 (1.02–3.54)	**0.044**	1.16
Chronic lung disease	1.02 (0.43–2.44)	0.959	‐	‐	
Dyslipidaemia	0.65 (0.33–1.31)	0.23	‐	‐	
Chronic liver disease	3.15 (0.92–10.81)	0.069	‐	‐	
Active malignancy	6.43 (1.86–22.27)	0.003	6.15 (1.79–21.12)	**0.004**	1.02
SOFA score (per 1‐point increase)	1.02 (0.91–1.14)	0.771	‐	‐	
Glasgow Coma Score (per 1‐point decrease)	1.01 (0.95–1.07)	0.815	‐	‐	
d‐Dimer (per 1 mg/L FEU increase)	1 (0.99–1.01)	0.8	‐	‐	
NLR (per 1‐point increase)	1.01 (1–1.02)	0.019	1.01 (1–1.02)	**0.016**	1.03
Platelets count (per 100 × 10^3^/µl decrease)	1.38 (1. 10–1.74)	0.005	1.41 (1.13–1.75)	**0.002**	1.05
Ferritin (per 500 µg/L increase)	1.05 (1.02–1.09)	0.003	1.05 (1.02–1.08)	**0.002**	1.04
CRP (per 10 mg/L increase)	1.01 (0.99–1.03)	0.199	‐	‐	
PaO_2_/FiO_2_ ratio (per 50 mmHg decrease)	1.13 (0.87–1.46)	0.37	‐	‐	
Bilirubin (per 10 μmol/L increase)	1.16 (1–1.35)	0.046	1.19 (1.04–1.36)	**0.01**	1.06
Creatinine (per 44.2 μmol/L increase)	1.01 (0.95–1.07)	0.866	‐	‐	

Abbreviations: BMI, body mass index; CRP, C‐reactive protein; GCS, Glasgow coma scale; NLR, neutrophil‐to‐lymphocyte ratio; PaO_2_/FiO_2_ ratio, arterial oxygen partial pressure divided by the fraction of inspired oxygen; VIF, variance inflation factor.

## 4 DISCUSSION

Despite a high per capita SARS‐CoV‐2 infection rate, Qatar has one of the lowest case fatality rates.^2^ In our analysis of the first 1079 critically ill COVID‐19 patients, overall in‐hospital mortality was 12.6%, while mortality in patients who required IMV support was 25.9%.

The mortality observed among critically ill COVID‐19 patients in Qatar is lower than the previous reports from other countries. Lower mortality was also observed for the subgroup of patients who required IMV. In China, initial studies of critically ill COVID‐19 patients reported high mortality of 61.5%.^8^, ^12^ Wang et al. also reported a higher mortality rate of 97% among invasively ventilated patients.^11^ In the USA, a mortality rate of 31%–50% in critically ill COVID‐19 patients and 36%–88% in patients who required IMV has been reported.^4^, ^5^, ^6^ In Europe, the reported mortality in critically ill COVID‐19 patients ranged from 32% to 49%.^13^, ^14^, ^15^ In a large randomized controlled trial that examined the benefit of dexamethasone for reducing mortality among COVID‐19 patients, mortality among patients receiving IMV was 29.3%.^16^ A systematic review and meta‐analysis of 24 studies, including 10,150 patients with complete ICU data, found a pooled ICU mortality rate of 41.6%.^17^

A possible explanation of the lower mortality in the current study might be the higher percentage of steroid usage since the initial phases of the pandemic, which was later proven beneficial by randomized controlled trials.^16^, ^18^ In the current study, 85.6% of patients received corticosteroids. Also, all cases that needed ICU admission were admitted irrespective of advanced age or underlying comorbidities such as advanced stage of malignancy. Our system had an adequate number of ventilators and respiratory therapists to manage them. Being able to provide patients with the appropriate treatments like bronchoscopies, continuous RRT, tracheostomies, advanced hemodynamic monitoring, and ECMO without delay has for many patients been the difference between life and death. However, further research is needed to explore whether genetic or environmental factors may explain such differences in disease outcomes.

In the current study, hypertension and diabetes mellitus were the most common comorbidities among critically ill COVID‐19 patients (47.3% and 42.6%, respectively). The most common comorbidities in previous studies were also hypertension and diabetes mellitus.^5^, ^10^, ^15^, ^16^, ^19^, ^20^ The association of these diseases with mortality varied among reported literature, with some studies suggesting systemic hypertension is significantly associated with mortality^11^, ^20^, ^21^, ^22^ and others associating diabetes with mortality.^23^, ^24^, ^25^ Despite being more common in the patients who died in our study cohort, hypertension and diabetes were not independently associated with higher mortality after adjustment for other confounders.

The incidence of secondary infections during ICU stay was high (39.3%) among our study population. It was highest among patients who received steroids and tocilizumab followed by patients who received corticosteroids only then patients who received tocilizumab only. Zangrillo et al. observed the same rate of secondary bacteremia (37%) in their study of 73 IMV patients.^20^ Although secondary infections can be explained by the use of corticosteroids and/or tocilizumab, few authors suggest that secondary infections occur in COVID‐19 because of the disproportionate compensatory anti‐inflammatory syndrome.^26^

In our study, the rate of confirmed VTE events was lower compared to previous studies (4.1% vs. 59%, respectively).^27^ On the other hand, around 8.9% of the study cohort developed bleeding during their ICU stay. Nine cases (9.4%) among them had fatal bleeding. These findings might be explained by the higher rate of using therapeutic anticoagulation based on d‐dimer levels according to our local protocol. Overall, venous thromboembolism, rhabdomyolysis, secondary infection, and bleeding were significantly higher among nonsurvivors than survivors (Table 4). These complications represent possible aetiologies of mortality among critically ill COVID‐19 patients in addition to fatal respiratory failure.

**Table 4:** Complications observed among the study cohort

Complication	Survivors (*n* = 943)	Nonsurvivors (*n* = 136)	*p* value	Total (*n* = 1079)
Venous thromboembolism	32 (3.4%)	12 (8.8%)	0.003	44 (4.1%)
Rhabdomyolysis	129 (13.7%)	60 (44.1%)	<0.001	189 (17.5%)
Secondary Infection	310 (32.9%)	114 (83.8%)	<0.001	424 (39.3%)
Bleeding	54 (5.7%)	42 (30.9%)	<0.001	96 (8.9%)

Our study has several strengths. To our knowledge, this is the first study to describe the characteristics and outcomes of critically ill COVID‐19 patients in the Middle East. The study sample size is large, representing four months of ICU admissions during peak stages of admission. We followed up with all patients to have complete outcome data of either in‐hospital death or hospital discharge.

However, we also acknowledge that the current analysis has several limitations. The study design did not allow us to analyze detailed ventilatory settings and measurements. Our study population is mostly men, limiting its generalizability to the entire population. Most patients received a combination of antiviral and antibacterial therapy, limiting our ability to study the effects of each agent independently.

## 5 CONCLUSIONS

The mortality rate among critically ill COVID‐19 patients is lower in Qatar compared to other countries, which might be attributed to several factors, including the demographic characteristics, low‐comorbidity burden, effective national health strategy, and response to the pandemic. Older age, CKD, active malignancy, higher neutrophil‐to‐lymphocyte ratios, lower platelet counts, higher serum ferritin levels, and higher serum bilirubin levels are independent predictors of in‐hospital mortality in these low fatality settings.

## ETHICS STATEMENT

This study was approved by the Medical Research Center of Hamad Medical Corporation (MRC‐05‐134). The study has been conducted in accordance with the ethical standards noted in the 1964 Declaration of Helsinki and its later amendments or comparable ethical standards. No consents were obtained due to the retrospective nature of the study.

## AUTHOR CONTRIBUTIONS

*Conceptualization, data curation, methodology, writing (original draft); writing (review and editing)*: Mohamad Y. Khatib, Dore C. Ananthegowda, Moustafa S. Elshafei, Hani El‐Zeer, Wael I. Harb Abdaljawad, Muhsen A. Shaheen, Abdulsalam S. Ibrahim, Ahmed A. Soliman, Ahmed S. Mohamed, Mohammad Al‐Wraidat, Amna Ahmed, Abdulqadir J. Nashwan, Mohamed O. Saad, Adeel A. Butt, Muna A. Al‐Maslamani, Ahmed Al‐Mohammed. *Formal analysis, writing (original draft); writing (review and editing):* Ahmad A. Abujaber. *Funding acquisition*: Mohamad Y. Khatib. All authors have read and approved the final version of manuscript A. J. N. had full access to all of the data in this study and takes complete responsibility for the integrity of the data and the accuracy of the data analysis. Dr. Mohamad Y. Khatib affirms that this manuscript is an honest, accurate, and transparent account of the study being reported; that no important aspects of the study have been omitted; and that any discrepancies from the study as planned (and, if relevant, registered) have been explained.

## Supporting information

Supplementary information.

## Data Availability

The authors confirm that the data supporting the findings of this study are available within the article and its supplementary materials.
