# Supplementary material for: Predictors of mortality and morbidity in critically ill COVID‐19 patients: An experience from a low mortality country
Source: Health Sci Rep. 2022 May 17;5(3):e542. doi: 10.1002/hsr2.542 (PMC9111769; doi:10.1002/hsr2.542)
Supplement: Supplementary file 1 — Supplementary information. [file HSR2-5-0-s001.docx]

Supplementary appendix 1: Early predictors of mortality among male patients only (n=1017)

| Predictors | Initial full model | | Final model | |  |
| --- | --- | --- | --- | --- | --- |
|  | OR (95% CI) | P value | OR (95% CI) | P value | VIF |
| Age  (per 10 years increase) | 2.29 (1.88 - 2.78) | <0.001 | 2.3 (1.91 - 2.77) | **<0.001** | 1.18 |
| BMI  (per 1 kg/m^2^) | 1.03 (0.99 - 1.08) | 0.179 | - | **-** |  |
| Diabetes | 1.05 (0.66 - 1.67) | 0.847 | - | **-** |  |
| Hypertension | 1.14 (0.70 – 1.88) | 0.596 | - | **-** |  |
| Coronary artery disease | 0.51 (0.26 -0.98) | 0.043 | 0.55 (0.29 – 1.01) | 0.054 | 1.14 |
| Chronic kidney disease | 1.66 (0.75 - 3.69) | 0.215 | 1.89 (0.97 - 3.67) | 0.061 | 1.11 |
| Chronic lung disease | 0.74 (0.27 – 1.99) | 0.548 | - | **-** |  |
| Dyslipidaemia | 0.89 (0.44 - 1.81) | 0.748 | - | **-** |  |
| Chronic liver disease | 2.78 (0.72 - 10.73) | 0.138 | - | **-** |  |
| Active malignancy | 8.97 (2.19 – 36.81) | 0.002 | 9.09 (2.25 – 36.71) | **0.002** | 1.01 |
| SOFA score  (per 1-point increase) | 1.00 (0.89 - 1.12) | 0.997 | - | - |  |
| Glasgow Coma Score  (per 1-point decrease) | 1.03 (0.96 - 1.1) | 0.445 | - | - |  |
| D-Dimer  (per 1 mg/L FEU increase) | 1 (0.99 - 1.02) | 0.635 | - | - |  |
| NLR  (per 1-point increase) | 1.01 (1 - 1.02) | 0.039 | 1.01 (1 - 1.02) | **0.020** | 1.03 |
| Platelets count  (per 100x10^3^/µL decrease) | 1.39 (1. 10 - 1.76) | 0.006 | 1.42 (1.13 - 1.77) | **0.002** | 1.04 |
| Ferritin  (per 500 µg/L increase) | 1.06 (1.02 - 1.10) | 0.003 | 1.06 (1.02 - 1.09) | **0.002** | 1.05 |
| CRP  (per 10 mg/L increase) | 1.01 (0.99 - 1.03) | 0.450 | - | - |  |
| PaO_2_/FiO_2_ ratio  (per 50 mmHg decrease) | 1.08 (0.83 - 1.41) | 0.570 | - | - |  |
| Bilirubin  (per 10 μmol/L increase) | 1.16 (0.99-1.34) | 0.059 | 1.18 (1.03 - 1.35) | **0.014** | 1.05 |
| Creatinine  (per 44.2 μmol/L increase) | 1.01 (0.95 - 1.07) | 0.773 | - | - |  |

BMI: body mass index; CRP: C-reactive protein, GCS: Glasgow coma scale, NLR: Neutrophil-to-lymphocyte ratio; PaO2/FiO2 ratio: arterial oxygen partial pressure divided by the fraction of inspired oxygen, VIF: variance inflation factor
